# A *gacS* Deletion in *Pseudomonas aeruginosa* Cystic Fibrosis Isolate CHA Shapes Its Virulence

**DOI:** 10.1371/journal.pone.0095936

**Published:** 2014-04-29

**Authors:** Khady Mayebine Sall, Maria Guillermina Casabona, Christophe Bordi, Philippe Huber, Sophie de Bentzmann, Ina Attrée, Sylvie Elsen

**Affiliations:** 1 INSERM, UMR-S 1036, Biology of Cancer and Infection, Grenoble, France; 2 CNRS, ERL 5261, Bacterial Pathogenesis and Cellular Responses, Grenoble, France; 3 UJF-Grenoble 1, Grenoble, France; 4 CEA, DSV/iRTSV, Grenoble, France; 5 Laboratoire d’Ingénierie des Systèmes Macromoléculaires, UMR 7255 CNRS - Aix Marseille University, Marseille, France; University of North Dakota, United States of America

## Abstract

*Pseudomonas aeruginosa*, a human opportunistic pathogen, is capable of provoking acute and chronic infections that are associated with defined sets of virulence factors. During chronic infections, the bacterium accumulates mutations that silence some and activate other genes. Here we show that the cystic fibrosis isolate CHA exhibits a unique virulence phenotype featuring a mucoid morphology, an active Type III Secretion System (T3SS, hallmark of acute infections), and no Type VI Secretion System (H1-T6SS). This virulence profile is due to a 426 bp deletion in the 3′ end of the *gacS* gene encoding an essential regulatory protein. The absence of GacS disturbs the Gac/Rsm pathway leading to depletion of the small regulatory RNAs RsmY/RsmZ and, in consequence, to expression of T3SS, while switching off the expression of H1-T6SS and Pel polysaccharides. The CHA isolate also exhibits full ability to swim and twitch, due to active flagellum and Type IVa pili. Thus, unlike the classical scheme of balance between virulence factors, clinical strains may adapt to a local niche by expressing both alginate exopolysaccharide, a hallmark of membrane stress that protects from antibiotic action, host defences and phagocytosis, and efficient T3S machinery that is considered as an aggressive virulence factor.

## Introduction

*Pseudomonas aeruginosa* is an opportunistic Gram negative bacterium able to trigger either severe acute or chronic human infections, depending on the environmental signals it encounters. The persistence of the bacterium during decades in the lungs of individuals with cystic fibrosis (CF) is associated with massive and inefficient inflammation contributing to airway epithelium destruction, decline of lung function and death due to respiratory failure [1]. During chronic infection, *P. aeruginosa* has to survive and adapt to the stressful environment encountered in the CF lungs where it is continuously exposed to antibiotics, oxidative and osmotic stresses as well as active host immune system. The CF respiratory mucus has been shown to directly impact bacterial gene transcription [2], [3]. A CF mucoid strain was reported to trigger, in response to the CF niche, the synthesis of enzymes protecting the bacteria against oxidative stress and the activation of genes encoding the HSI-I Type VI Secretion System (H1-T6SS), known to play a role in bacterial competition [4], [5], [6]. In addition, the alginate production was unexpectedly repressed, and the expression of two small RNAs (PA2G_05393.1 and PA2G_03487.1) with putative regulatory roles was observed, pointing out the major effect of contact with CF mucus on bacterial physiology [3].

Besides these immediate adaptive responses, *P. aeruginosa* is also prone to accumulate point mutations and/or significant genomic rearrangements induced by extrinsic and intrinsic factors associated with CF chronic disease [1], [7]. Mutations in global regulatory genes, such as *lasR*, *vfr*, *rpoS*, or other regulatory genes such as *mucA*, are frequently observed in CF isolates and these mutations profoundly affect virulence gene expression [1], [8]. The most obvious phenotypic changes of *P. aeruginosa* observed during infection in CF disease are conversion to mucoidy, emergence of Small Colony Variants (SCV), acquisition of antibiotic multi-resistance, loss of motility and shutdown of quorum sensing (QS) system [7], [9]. Conversion to mucoidy is proposed to be a major survival mechanism promoting the persistence of the bacterium in CF lungs; it mostly results from the over-expression of the alginate polysaccharide by the alternative sigma factor AlgU, desequestrated from its anti-sigma partner MucA due to mutations in *mucA* [10], [11]. More aggressive virulence factors secreted by Type II (T2SS) and Type III (T3SS) secretion machineries are switched off, thus leading to CF-adapted strains usually less virulent in animal models than the primary infecting ones [9]. However, while sharing some generally-accepted traits, adaptive evolution and resulting phenotypes of CF isolates can be extremely diverse, even within isolates from the same CF patient sputum sample [1], [9], [12], [13].

The CHA strain is a good example of *P. aeruginosa* multifaceted adaptation in CF disease. It was originally isolated from a CF patient [14], 4 years after the first airways colonization by *P. aeruginosa*, and is thus not considered as an early infecting strain. This is an O6 serotype strain belonging to clone J, one common clonal group disseminated worldwide [15]. CHA is mucoid [16], a hallmark of chronicity, but at the same time, it is a highly efficient T3SS effector-producer fitting with acute infectious status [17], [18], [19], [20]. Very recently, CHA has been included in the international *P. aeruginosa* reference panel, stressing the relevance to study its pathogenicity [21].

Using phenotypic and complementation experiments in parallel to genome analysis, we report in the present study that virulence properties of CHA result from an intrinsic genetic deletion leading to the absence of the histidine kinase (HK) GacS. GacS/GacA is a two-component regulatory system (TCS) that controls transcription of two small regulatory RNAs (sRNAs), RsmY and RsmZ. These two sRNAs prevent RsmA binding to its mRNA targets and consequently modulate, directly or indirectly, approximately 500 genes belonging to the RsmA regulon [22], [23], [24]. Activity of GacS is regulated in an opposite manner by two inner membrane sensors, RetS and LadS, in response to still unidentified stimuli [25], [26]. The RetS/LadS/Gac/Rsm cascade is known to be a master regulator of the virulence factors of *P. aeruginosa*, controlling the switch in their expression during the acute/chronic phase transition (reviewed in [27]). Hence, as illustrated here, mutations in this regulatory pathway profoundly impact the global virulence traits.

## Materials and Methods

### Bacterial strains

The *P. aeruginosa* and *Escherichia coli* strains, as well as the plasmids used in this study, are listed in Table 1. The genotype of the CHA strain was determined using the ArrayTube genotyping method [15]. Cells were grown aerobically in Luria Bertani (LB) medium at 37 °C with agitation. *P*. *aeruginosa* was also cultured on Pseudomonas Isolation Agar plates (PIA; Difco). Antibiotics were added at the following concentrations (in µg/ml): 100 (ampicillin), 25 (gentamycin), 25 (kanamycin) and 10 (tetracyclin) for *E. coli*, 500 (CHA) or 200 (PAO1) (carbenicillin), 400 (CHA) and 200 (PAO1) (gentamycin) and 200 (tetracycline) for *P. aeruginosa*.

**Table 1:** Bacterial strains and plasmids used in this work.

Strain or plasmid	Relevant characteristics	Source/reference
*P. aeruginosa*		
PAO1	Wound isolate, sequenced laboratory strain	J. Mougous
TB (TBCF10839)	Cystic fibrosis (CF) isolate	[28]
LES400	CF epidemic strain	[29]
KK1	CF isolate	[30]
CF6	CF isolate	[17]
PA7	Wound isolate	[31]
PAK	Clinical isolate	D. Bradley
CHA	Mucoid CF isolate	[14]
CHA-GacS	CHA with wild-type PAO1 *gacS* in the chromosome	This study
CHAΔ*rsmA*	CHA deleted of the *rsmA* gene	This study
CHAΔ*retS*	CHA deleted of the *retS* gene	This study
CHAΔ*retS*-GacS	CHAΔ*retS* with PAO1 *gacS* in the chromosome	This study
Plasmids		
pCR-Blunt II-TOPO	Kn^r^; commercial cloning vector	Invitrogen
pEX100-T	Ap^r^; mobilisable vector, non-replicative in *P. aeruginosa*	[32]
pRK2013	Kn^r^; helper plasmid with conjugative properties	[33]
pUC18-mini-Tn7T-	Gm^r^; translational fusion vector	
lacZ20-Gm		[34]
pUC18-mini-Tn7T-	Promoter and entire *gacS* sequence of PAO1 cloned into	
pGacS	the integrative vector	This study
pUC18-miniTn7T-	*pfha1-lacZ* translational fusion in pUC18-mini-TN7T-	
PA0081-lacZ	lacZ20-Gm	[23]
pCTX-PA0081-lacZ	Tc^r^; *pfha1-lacZ* transcriptional fusion in mini-CTX-lacZ	[23]
pJN105	Gm^r^; *pBAD* transcriptional fusion vector	[35]
pJN-GacS	*pBAD*-*gacS* transcriptional fusion in pJN105	This study
pVLT31	Tc^r^; *plac* transcriptional fusion vector	[36]
pVLT-RsmA	*plac-rsmA* transcriptional fusion in pVLT31	This study
pMP220	Tc^r^; *lacZ* transcriptional fusion vector	[37]
pMP220-*rsmY*-*lacZ*	*prsmY-lacZ* transcriptional fusion in pMP220	[38]
pMP220-*rsmZ*-*lacZ*	*prsmY-lacZ* transcriptional fusion in pMP220	[38]

### Animals

All protocols in this study were conducted in strict accordance with the French guidelines for the care and use of laboratory animals. The protocol for mouse infection was approved by the animal research committee of the institute (CETEA: Comité d' ÉThique en Expérimentation Animale). Pathogen-free BALB/c male mice (8-10 weeks) were obtained from Harlan Laboratories and housed in the CEA animal care facilities.

### Genetic constructions

Deletion of *rsmA* and *retS* – Fused uspstream and downstream flanking regions of *rsmA* and *retS* were amplified by Splicing by Overlap Extension-Polymerase Chain Reaction (SOE-PCR) procedure using appropriate primer pairs (Table S1). The resulting fragments of 846 bp and 834 bp, respectively, were cloned into pCR-Blunt II-TOPO vector, sequenced and then subcloned into the *Sma*I site of the suicide plasmid pEX100-T. The resulting pEXΔRsmA and pEXΔRetS plasmids carry the counter-selectable *sacB* marker from *Bacillus subtilis*, which confers sensitivity to sucrose. Both plasmids were mobilized into *P. aeruginosa* strain by triparental mating, using the conjugative properties of the helper plasmid pRK2013. Co-integration events were selected on PIA plates containing carbenicillin. Single colonies were then plated on PIA medium containing 5% (w/v) sucrose to select for the loss of plasmid: the resulting sucrose-resistant strains were checked for carbenicillin sensitivity and for *rsmA* or *retS* (wild-type or deleted gene) genotype by PCR.

Fusion of VSV-G epitope to GacS - The VSV-G-Tag-coding sequence was fused to the 3′ end of chromosomal PAO1 *gacS* or to that of gacS*, the CHA naturally truncated *gacS* gene, using SOE-PCR strategy. Upstream and downstream flanking regions of *gacS* and gacS* were PCR amplified using PAO1 or CHA genomic DNA, respectively, and appropriate primer pairs (see Table S1). The resulting fragments of 813 bp and 752 bp, respectively, were cloned into pCR-Blunt II-TOPO vector, sequenced, then inserted into the *Sma*I site of pEX100-T, leading to pEX-GacS-VSV-G and pEX-GacS*-VSV-G plasmids. Allelic replacement was performed as described above. The presence of VSV-G-encoding sequences in the PAO1 and CHA genomes was assessed by PCR and confirmed by sequencing of PCR fragments.

Complementation - The *rsmA* and *gacS* genes were PCR amplified from PAO1 genomic DNA using appropriate primer pairs (Table S1). The PCR products were cloned into pCR-Blunt II-TOPO and sequenced. The 0.23-kb *Xba*I-*Hin*dIII restriction fragment containing *rsmA* was cloned in pVLT31 plasmid under control of the IPTG-inducible *ptac* promoter. The 2.85-kb *Sma*I-*Xba*I restriction fragment containing *gacS* was cloned into pJN105 plasmid under the arabinose-inducible *pBAD* promoter. The two expression vectors were introduced in *P. aeruginosa* by transformation [39].

An integrative plasmid was also constructed to complement *gacS* mutation with one copy of the gene driven by its own promoter. A region comprising the 245 bp sequence upstream from *gacS* and the entire gene was PCR amplified from PAO1 genomic DNA using appropriate primers (Table S1). After sequencing, the *Sma*I-*Hin*dIII restriction fragment was cloned into pUC18-miniTn7T-Gm-LacZ20. The resulting plasmid was electroporated into the CHA strain along with the pTNS2, as described [34], leading to the CHA-GacS strain.

### β-Galactosidase assays

β-Galactosidase activity was assayed as already described [40].

### T3SS-dependent cytotoxicity assay

Bacteria were grown in LB to an *A*_600_ 1.0 and added to the macrophage cell line J774 (J774A.1, catalog No. TIB-67, ATCC) at a multiplicity of infection (MOI) of 5. Cell death was assessed at 3 h post-infection by using a cytotoxicity detection kit (lactate dehydrogenase LDH; Roche) as described [17].

### Sample preparation, antibodies and immunoblot analysis

H1-T6SS Hcp1 (for haemolysin-coregulated protein 1) production and secretion were assessed as described [41]. For GacS-VSV-G and GacS*-VSV-G analysis, 100 µl of cultures at *A*_600_ of 2.8-3.0 were harvested and analyzed as the total bacterial fraction. Membrane fractions were recovered after lysozyme treatment, sonication and ultracentrifugation. The samples were submitted to SDS-PAGE and immunoblotting analysis.

The following antibodies were used: polyclonal antibodies anti-Hcp1 already described [41]; polyclonal antibodies anti-VSV-G (Sigma Aldrich); monoclonal antibodies anti-RpoA (Neoclone); anti-Opr86 polyclonal antibodies, obtained after immunization of rabbits with HIS-Tev-o-Opr86 (G629-N752) and used at 1∶2000. The commercial secondary antibodies (anti-rabbit-HRP, anti-guinea pig-HRP, anti-mouse-HRP) were used as recommended by the manufacturers.

### Motility assays

Motilities were assayed on media as described [42]. All plates were inoculated with bacteria from overnight cultures on LB agar using sterile toothpicks.

### Air-liquid biofilm

The *P. aeruginosa* adherence assay was performed in individual glass tubes as previously described [43].

### RT-PCR and RT-qPCR

The strains were grown at 37°C under agitation in LB, that was supplemented with 5 mM EGTA and 20 mM MgCl_2_ (conditions of *in vitro* T3SS induction) for Reverse Transcriptase (RT)-qPCR analysis. Total RNA was either extracted with the TRIzol Plus RNA Purification Kit (Invitrogen) then treated with DNase I (Amplification Grade, Invitrogen), or the PureYield RNA Midiprep System (Promega), cleaned up and concentrated using the RNeasy kit (Qiagen). Yield, purity and integrity of RNA were further evaluated on Nanodrop and by agarose gel migration. Complementary DNA synthesis was carried out with SuperScript III First-Strand Synthesis System (Invitrogen) in presence or not of the SuperScript III RT enzyme to assess the absence of genomic DNA. For RT-PCR experiments, performed to assess the presence of GacS mRNA, PCR amplifications were performed using *Taq*PCRx DNA Polymerase (Invitrogen) and following the Basic PCR protocol described by the manufacturer. Calibration of the PCR amplification steps was done by varying number of cycles with primers targeting either *gacS* or 16S rRNA as a reference transcript. The RT-qPCR runs, used to quantify the effect of GacS on its target genes, were carried out on a CFX96 Real-Time System (Bio-Rad). Cycling parameters of the real time PCR were 98 °C for 2 min, following by 45 cycles of 98°C for 5 s and 60°C for 10 s, ending with a melting curve from 65 °C to 95 °C to assess the specificity of the amplification. To determine the amplification kinetics of each product, the fluorescence derived from the incorporation of EvaGreen into the double-stranded PCR products was measured at the end of each cycle using the SsoFast EvaGreen Supermix 2X Kit (Biorad). The results were analyzed using the Bio-Rad CFX Manager Software 3.0 (Bio-Rad). The relative mRNA quantity of each gene under GacS production as compared to absence of production was analyzed using the Relative Expression Software Tool REST2009 (Qiagen) with a pair wise fixed reallocation randomization test [44] coupled to a standard error (SE) calculated via a Taylor algorithm. The 16S rRNA was used as reference for normalization. The sequences of all primers are given in Table S1.

## Results

### H1-T6SS expression is off in CF strain CHA

The mucoid CF isolate CHA has been previously reported as harboring T3SS-dependent cytotoxicity *in vitro* [45], [46]. As CF strains usually exhibit a decreased toxicity compared to strains triggering acute infection [9], we compared the virulence of CHA in mice to that of the PAO1 reference strain, which is a wound isolate. The CHA strain was clearly more virulent than the PAO1 strain in a murine acute model of lung infection (Figure S1A); this was associated with an increased dissemination of the bacteria both in blood and spleen compared to PAO1 (Figure S1B). The CHA T3SS-deficient strain exhibited a reduced toxicity in the model (data not shown) which is in agreement with a previous report pointing to T3SS being a major actor in CHA virulence [18].

**Figure 1:**
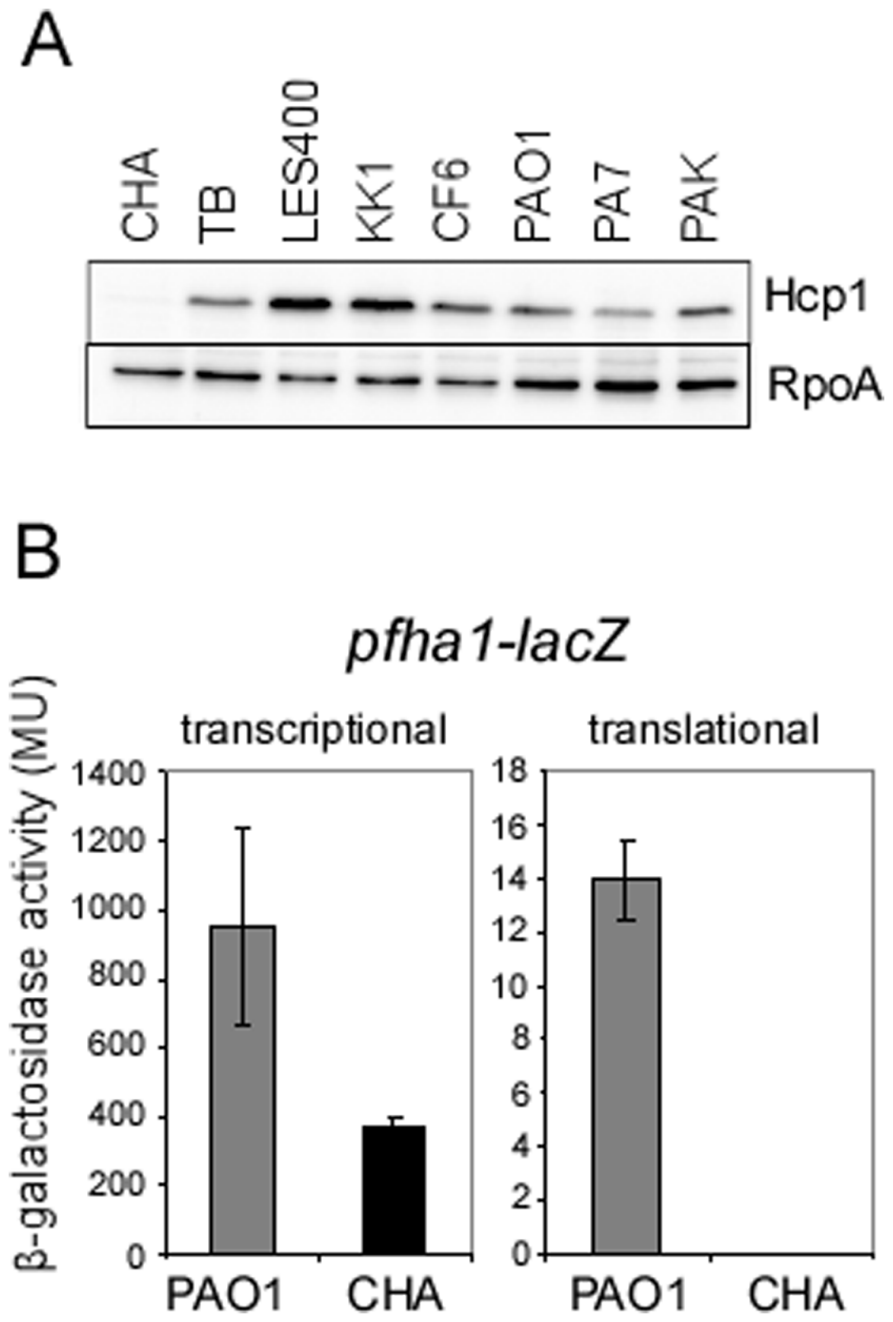
H1-T6SS is not expressed in the CHA strain. (A) Western blot analysis of Hcp1 from different *P. aeruginosa* CF and non-CF strains. The cytoplasmic RpoA protein is used as a loading marker. (B) β-galactosidase activities measured at *A*_600_ of 1.5 from PAO1 and CHA strains containing either a transcriptional or translational *pfha1*-*lacZ* fusions, as indicated. The bars indicate the standard deviations.

We then examined the expression of H1-T6SS (HSI-I), a machinery expressed during chronic infection [4], [5] and secreting bacterial toxins used for competing with other species in biofilm communities. Surprisingly, H1-T6SS production was undetectable in CHA as compared to other strains, notably CF strains TB, LES400, KK1 and CF6, or reference strains PAO1, PA7 and PAK, as assessed by the production of the Hcp1 component of the H1-T6SS apparatus (Figure 1A). The transcriptional fusion *fha1-lacZ* (*PA0081*) was expressed in CHA, albeit 2.4 fold less than in PAO1, while the corresponding translational fusion exhibited no reporter activity in CHA (Figure 1B). These results strongly suggested that CHA is incapable of H1-T6SS synthesis due to a default of mRNA stability and/or protein translation rather than an absence of HSI-I gene transcription.

### Levels of RsmA and RsmY/Z are perturbed in CHA

**Figure 2:**
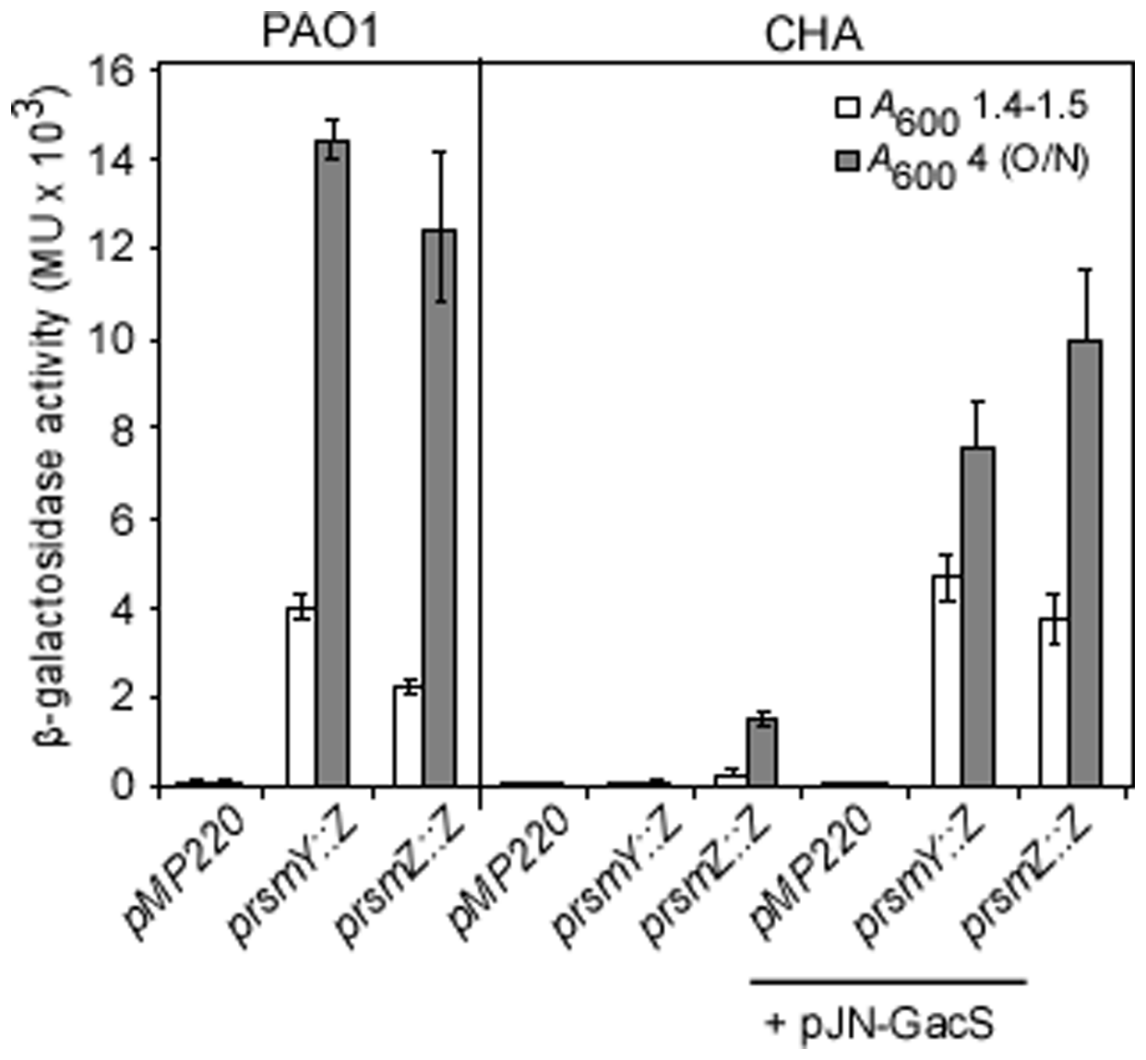
Expression of the regulatory sRNAs is affected in the CHA strains. The expression of *prsmY::lacZ* and *prsmZ::lacZ* was measured in PAO1 and CHA at two different *A*_600_. When indicated, the pJN-GacS plasmid was introduced. The plasmid pMP220 is the promoter-free *lacZ* plasmid used as a control. The reported values for enzyme activities are the average of at least two independent experiments performed in triplicate. The bars indicate the standard deviations. O/N: overnight culture.

**Figure 3:**
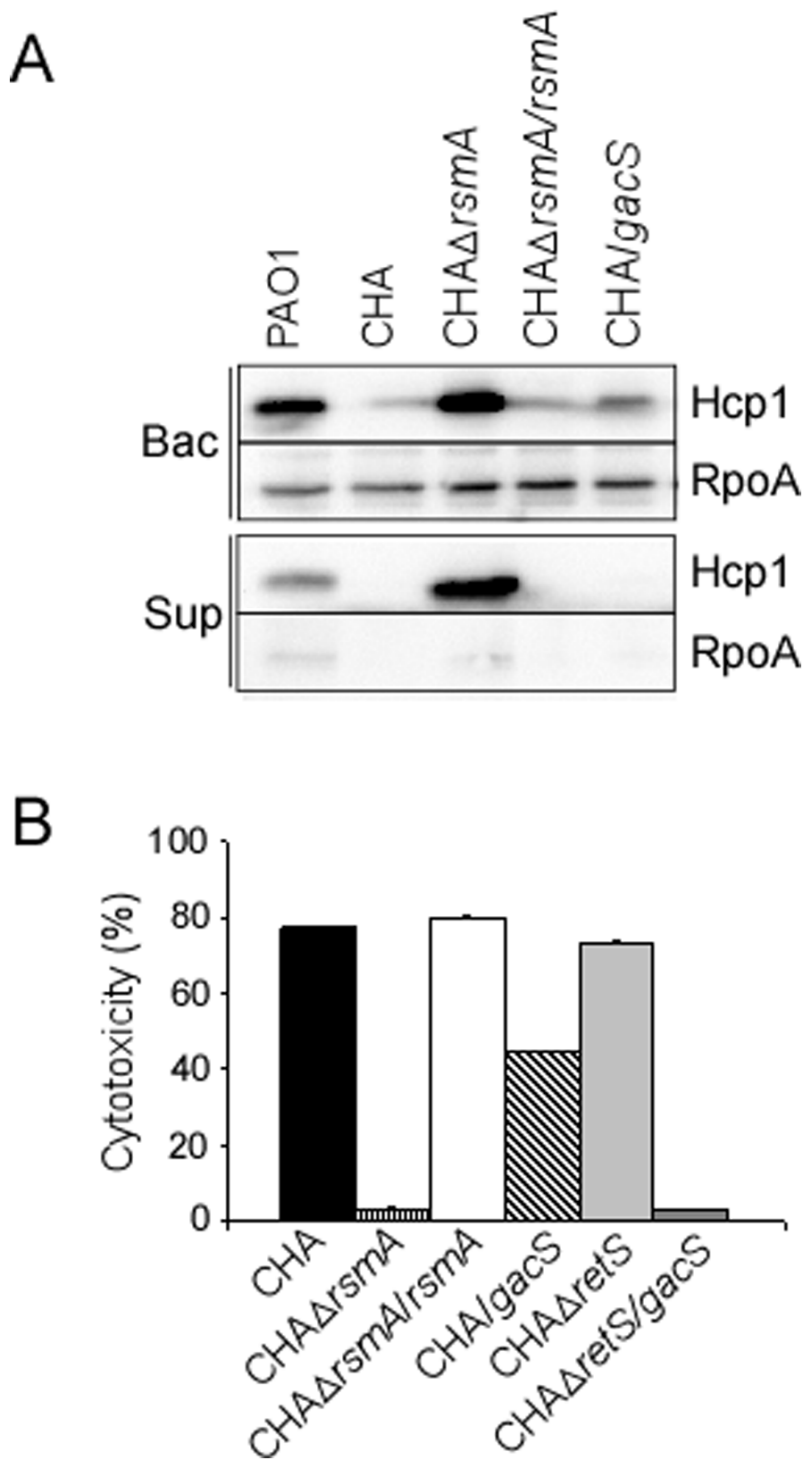
RsmA is responsible for absence of H1-T6SS and high T3SS activity in CHA. (A) Hcp1 synthesis and secretion were analysed by Western blot in the different strains as indicated. The cytoplasmic RpoA protein was used as a loading marker. Bac: bacteria, sup: supernatant. (B) T3SS-dependent cytotoxicity on J774 macrophages of wild-type, mutants and plasmid-complemented strains. Cytotoxicity was measured after 3 hours of infection and is expressed as a percentage of the total amount of LDH released from cells lysed with 1% Triton X-100. All tests were performed in triplicate.

T3SS and the H1-T6SS are known to be modulated in an opposite manner by the translational regulator RsmA, whose activity is antagonized by the two sRNAs, RsmY and RsmZ [23], [25]. Thus, we examined the *rsmY* and *rsmZ* expression in CHA. The *rsmY* gene was not expressed in CHA, and only a slight expression of *rsmZ* was observed, mainly in overnight culture (Figure 2), while in PAO1, the two sRNAs were efficiently transcribed, with higher level of *rsmY* expression compared to that of *rsmZ* (Figure 2), as already reported [3], [24]. These data strongly suggested that, in CHA strain, amounts of RsmY and RsmZ sRNAs were not sufficient to efficiently titrate RsmA and to relieve its effect on its target mRNAs. Indeed, high amounts of Hcp1 were produced in the supernatant and whole cell extracts of the CHA*ΔrsmA* mutant, a phenotype corrected by introduction of the *rsmA* wild-type gene *in trans* (Figure 3A). Additionally T3SS activity, assessed either by *in vitro* calcium-induced secretion of translocator proteins (data not shown) or T3SS-dependent cytotoxicity on macrophages (Figure 3B), was completely abolished by *rsmA* deletion and further re-induced by *rsmA* complementation.

Taken together, the absence of H1-T6SS synthesis in the CHA strain, resulting from a constant translational inhibition exerted by RsmA on H1-T6SS mRNAs, and the highly active T3SS, due to RsmA-mediated positive post-transcriptional regulation of ExsA expression [47], are both related to a defective *rsmY/Z* gene expression.

### A 426 bp chromosomal deletion led to a truncated and unstable GacS

**Figure 4:**
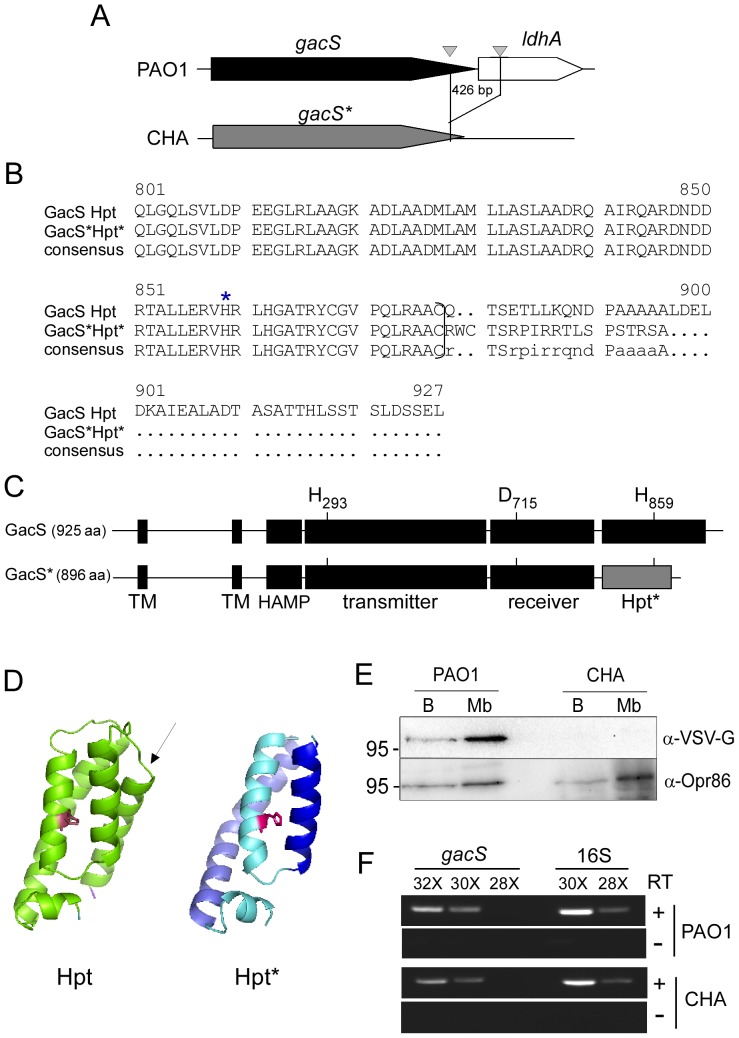
A genomic deletion in CHA affects the GacS regulator. (A) Genetic organization of *gacS* (PA0928, 2778 bp) and *ldhA* (PA0927, 990 bp) in PAO1. A 426 bp deletion in the CHA chromosome leads to 3′ truncated gacS* and 5′ truncated *ldhA* gene. The two 11-tandem imperfect repeats located into the PAO1 genes are depicted as arrowheads. (B) Sequence alignment of the Hpt (Histidine phosphotransfer) and Hpt* (truncated domain) domains of PAO1 GacS and CHA GacS*, respectively. The 48 last amino acids of GacS Hpt are replaced by unrelated 19 amino acids in Hpt*, at the position indicated by brackets. The star points to the conserved phosphorylated Histidine 859 residue. (C) GacS* contains all the phosphorylation sites but lacks the C-terminal part of Hpt domain. The length in amino acids (aa) of the proteins is indicated in brackets. TM: transmembrane helix. (D) Modeling of Hpt and Hpt* domains of GacS and GacS*, respectively, using PyMol. One of the helices of the four-helix bundle motif featuring the Hpt domain, that is indicated by an arrow, is missing in the predicted Hpt* domain. (E) Western blot analysis of GacS-VSV-G and GacS*-VSV-G in whole bacteria (B) and in membrane fractions (Mb) of PAO1 and CHA. The blots were developed by antibodies specific to the VSV-G epitope and to the porin Opr86 (PA3648) (loading control) as indicated. Note the absence of GacS*-VSV-G. Localisation of a molecular weight marker is indicated at the left (in kDa). (F) RT-PCR analysis of *gacS* gene transcript in PAO1 and CHA. The numbers of PCR cycles are indicated. 16S rRNA (16S) transcript levels indicate equal loading. Samples lacking SuperScript III RT enzyme during RT step (- RT) show that no DNA contamination was present.

We then investigated other regulatory players that could be involved in this deregulation by examining the recently sequenced genome of the CHA strain [48]. Indeed, a 426 bp deletion was found in the chromosome that affects the 3′ end (last 146 nucleotides) of *gacS* gene and the 5′ end (first 278 nucleotides) of the downstream gene *ldhA*, encoding a lactate dehydrogenase (Figure 4A) [48]. This *gacS* deletion generates a putative truncated protein that we named GacS*, possessing 19 unrelated residues in place of the 48 C-terminal residues of the PAO1 GacS protein (Figure 4B). The three phosphorylation sites (residues His-293, Asp-715 and His-859) are conserved (Figure 4C) but the fifth helix of the alternative transmitter domain (H2), also called Histidine PhosphoTransfer (Hpt) domain, is missing (Figure 4D).

To test whether this deletion impacts the stability and/or functionality of GacS*, we first provided *in trans* the Hpt domain (H2 domain) of PAO1 GacS protein [49], which did not complement the GacS* function (not shown). Thus we assessed by immunoblot the presence of the GacS and GacS* proteins in PAO1 and CHA, respectively. To do so, we first added at the 3′ end of the endogenous genes a sequence encoding the VSV-G Tag, generating GacS-VSV-G and GacS*-VSV-G proteins (see Materials and Methods). GacS*-VSV-G could not be detected in CHA, neither in the membranes nor in the whole cells, although GacS-VSV-G was clearly present in the membranes of the PAO1 strain (Figure 4E). We also ruled out the possibility that the absence of GacS* was a consequence of a transcription defect or an effect of the genomic deletion on mRNA stability by comparing the level of *gacS* transcripts in both PAO1 and CHA strains using RT-PCR (Figure 4F). Altogether, these results strongly suggested that truncation of GacS C-terminus in CHA led to an unstable protein.

**Figure 5:**
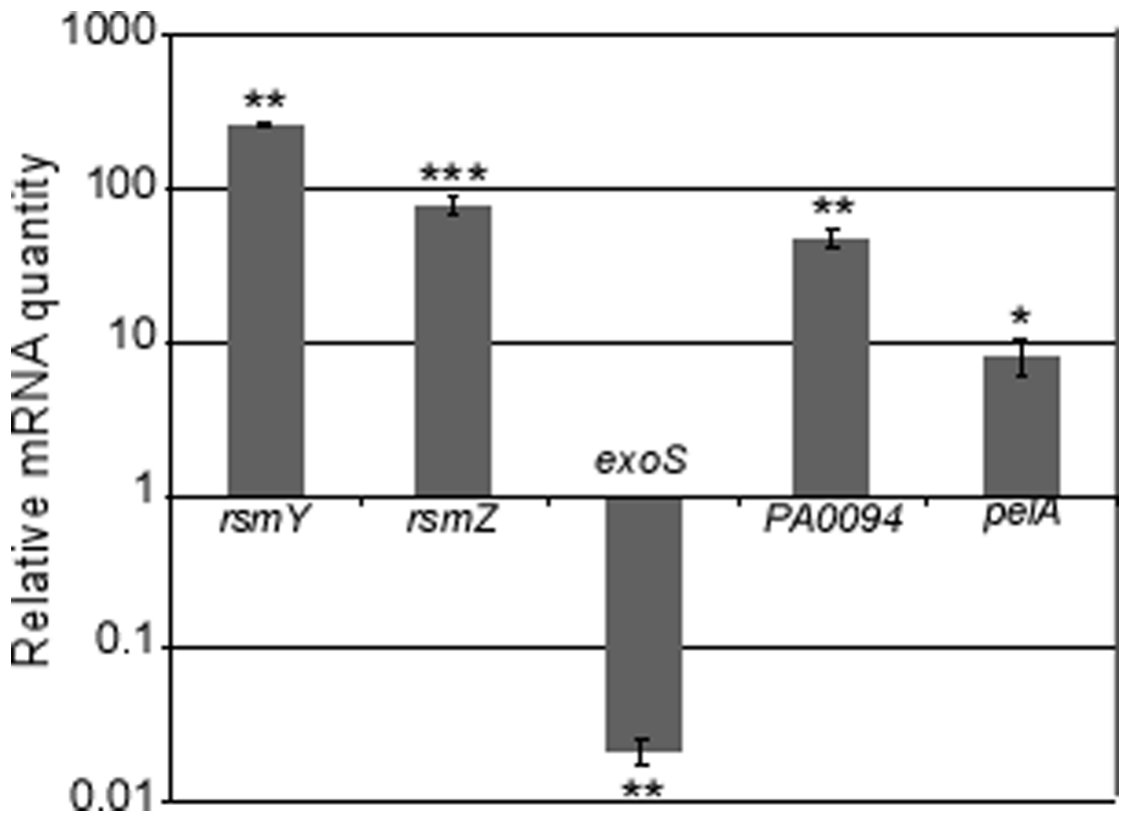
Complementation of CHA with *gacS* impacts sRNAs transcription as well as amount of major RsmA-target mRNAs, as assessed by RT-qPCR analysis. Relative mRNA quantity compares CHA/pJN-GacS to CHA/pJN105. Positive values indicate that mRNA levels are higher in *gacS*-complemented CHA. The p-values were calculated with REST2009 using Pair Wise Fixed Reallocation Randomisation Test. *: p<0.025, **: p<0.0015; ***: p<0.0002.

We next provided the PAO1 *gacS* gene cloned under the control of the *pBAD* promoter to the CHA strain. Introduction of the functional *gacS* gene increased the amounts of *rsmY* and *rsmZ* transcripts in CHA as assessed by RT-qPCR (Figure 5), data corroborated by the restoration of *rsmY/Z* promoter activity (Figure 2). It also increased quantity of *PA0094* transcripts, *PA0094* being a part of the H1-T6SS-encoding cluster (Figure 5), as well as the synthesis of Hcp1 (Figure 3A). In addition, providing *gacS* gene to CHA strain significantly reduced the level of *exoS* transcription, thus affecting the T3SS-dependent cytotoxicity on J774 macrophages (Figure 3B). Furthermore, even though *retS* deletion in CHA did not affect T3SS activity (due to absence of a functional GacS protein), introducing *gacS* in the CHAΔ*retS* background abolished it completely (Figure 3B). These features indicate that absence of a functional GacS may allow CHA to escape from the negative control exerted by GacS/GacA TCS on T3SS during chronic infection.

Taken together, these results demonstrate that the truncation of GacS in CHA is responsible for defect in *rsmY/Z* gene expression as well as for alterations observed in T3SS and H1-T6SS synthesis.

### The physiological absence of GacS shapes the phenotype of the clinical isolate

To get an overview of the impact of GacS absence in CHA, we investigated some of the major phenotypic traits of the bacterium.

**Figure 6:**
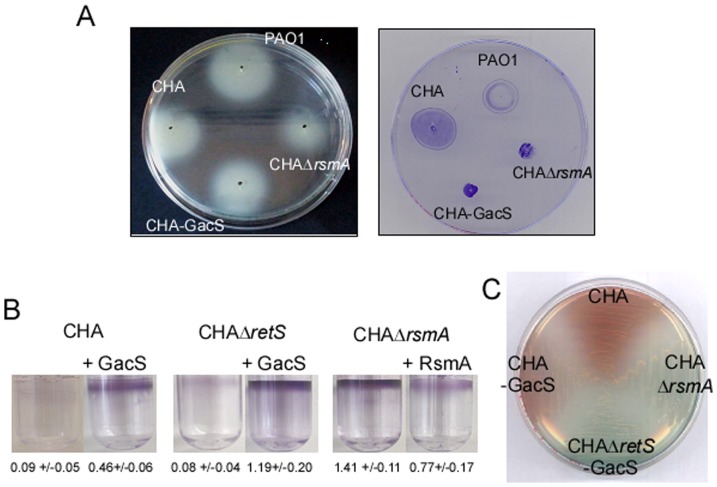
Impact of *gacS* inactivation in CHA on different virulence traits. (A) Swimming (left) and twitching (right) motilities of indicated strains. CHA-GacS contains the *gacS* gene amplified from PAO1 integrated into the chromosome. The images of the plates with regard to both the extent of spreading and morphology of motile bacteria are representative. (B) Air-liquid biofilm assay of indicated strains, containing either the empty plasmid (control) or the plasmid expressing the protein of interest, as indicated. For the assays with GacS, 0.5% arabinose was added in the medium. Biofilm was stained with crystal violet after 24 h at 30°C and quantified. The means of each assay performed in quadruplicate and their standard deviation are indicated below the corresponding pictures. (C) Pigment production in CHA is strongly affected by *rsmA* mutation, and *retS* mutation when *gacS* is integrated into the chromosome, as observed on PIA plate.

At first we studied its swimming and swarming motilities [42], as RsmA positively affects genes involved in formation of Type IVa pili and function of the flagellum [22]. These extracellular appendages are fundamental for motility, biofilm formation, and efficient injection of T3SS toxins into eukaryotic cells as this process requires cell adherence [50], [51], [52], [53]. CHA clearly possesses functional flagellum and Type IVa pili as it is able to swim comparably to PAO1 and exhibits even better twitching motility (Figure 6A). As expected, *rsmA* mutation in CHA resulted in a decrease in the two motilities. Introduction of a functional copy of *gacS* in the chromosome of CHA did not substantially change its swimming motility, and only twitching was affected and down-regulated in our laboratory conditions (Figure 6A). Thus, unlike frequently reported for CF-adapted strains [7], CHA is a motile CF isolate, with functional flagellum and Type IVa pili that may contribute to its cytotoxic phenotype.

Since synthesis of Pel and Psl exopolysaccharides is known to be under the control of the Gac/Rsm post-transcriptional cascade and to mediate biofilm formation [25], [53], we examined the ability of CHA and different mutants to form biofilm and, besides constitutive alginate production, synthesize Pel exopolysaccharide. CHA did not form biofilm rings in static growth conditions and inactivation of *rsmA* gene in CHA led to a hyperbiofilm phenotype (Figure 6B). Introduction of a wild-type *gacS* gene *in trans* restored the ability of CHA to produce biofilm and triggered an 8.3 increase in *pelA* transcript level (Figure 5), while it further enhanced biofilm production in a CHAΔ*retS* strain (Figure 6B).

Interestingly, different from the blue-green color of PAO1, the CHA strain exhibits a reddish color on PIA plate, likely resulting from a diffusible molecule (Figure 6C). This phenotype was not due to pyomelanin production, since inactivation of the *hpd* gene encoding the enzyme synthesizing HGA pyomelanin precursor [54] did not affect pigmentation (not shown). The production of this pigment is clearly under control of the Gac/Rsm pathway, since inactivation of *rsmA* abolished the red pigmentation to a level comparable to the one observed when wild-type *gacS* was introduced in a CHAΔ*retS* background (Figure 6C). Complementation of CHA with *gacS* gene affected the phenotype slightly. These data indicated that production of this red pigmentation, similar to T3SS, should escape from the control of Gac/RsmYZ during chronic infection. It is known that RsmA controls synthesis of pigments like pyocyanin [22], [55], and *P. aeruginosa* is able to synthesize other phenazines, as two red pigments called aeruginosins A and B, whose biological relevance is still not known [56]. Therefore the red color may rely on specific combination and quantities of these pigments. It is important to point out that phenazines are pigments with redox properties that contribute to bacterial pathogenicity in both acute and chronic models of infection. As identity and amount of phenazines are crucial for toxicity as well as for biofilm formation ([57] and references herein), as illustrated for the precursor of pyocyanin called 5MPCA which is more efficient for yeast killing than pyocyanin itself [56], identifying the nature of the Gac/Rsm pathway-regulated pigment(s) produced by CHA could give new insights in the physiological impact of this pigmentation.

All these data indicated that a deletion in *gacS* gene shapes pathogenic ability of the CHA clinical isolate by blocking the Gac/Rsm pathway. Indeed, CHA is a mucoid and motile strain expressing acute virulence factors that has lost the ability to switch from acute to chronic lifestyle.

## Discussion

Particular environmental conditions found in chronic infection, such as in CF, force *P. aeruginosa* to adapt in order to survive. In this work, by combining genomic and extensive phenotypic analyses, we found that an intrinsic genomic deletion in the CF strain CHA equips bacteria with virulence factors that are considered to be more aggressive and are in general associated with acute infections. Indeed, one of the key regulatory pathways, the RetS/LadS/Gac/Rsm pathway, proposed to control the transition between acute and chronic infection-associated phenotypes [25], [26], is impaired by a genomic deletion that affects the *gacS* gene region leading to an unstable truncated form of GacS. The same signalling pathway was found affected by mutations either in *ladS* [58] or in *retS* [59] in two other clinical strains isolated from acute and chronic infection, respectively. However, inactivation of GacS, the master regulator of this pathway, impacts more strongly the read-outs than that of LadS and RetS, by blocking the switch orchestrated by the Gac/Rsm pathway.

Spontaneous mutations in *gacS* and/or *gacA* represent the most important mechanism responsible for phenotypic variation of many pseudomonads, as observed in rhizosphere-associated *Pseudomonas* [60]. *Pseudomonas* sp. strain PCL1171 undergoes phase variation which is characterized by different production of secondary metabolites and exoenzymes. This phenotypic variation was reported to be caused by spontaneous and reversible mutations in *gacS* and *gacA* genes, probably randomly generated by an inefficient MutS-dependent repair of replication-related mismatches [60], [61]. One of these *gacS* mutations, a 307 bp deletion event, implied beforehand a spontaneous mutation that created a perfect 10 bp-direct tandem repeat allowing the recombination rearrangement [61]. A similar mechanism might have occurred in CHA genome to generate the deletion between the two 11-tandem imperfect repeats (5′-CGGCCTGCCA/GG) flanking the 3′ end of the *gacS* gene and the 5′ end of the *ldhA* gene in PAO1 genome (Figure 5*A*).

Numerous *P. aeruginosa* phenotypic variants are retrieved from the CF lungs, as the genome constantly accumulates mutations [1], [7] and even short-term growth in biofilm was shown to generate high genetic diversity in *P. aeruginosa* communities [62]. Interestingly, *gacS* mutants in PA14 are prone to generate stable SCVs when growing in biofilm or exposed to stresses [63] as well as *in vivo* [64]. When CHA was grown in static conditions *in vitro*, we also observed the emergence of stable SCV-like colonies (not shown). These observations suggest that the absence of GacS might have conferred to the CHA bacterium further advantage for persistence in CF lung by providing it with the capacity to convert to stress-tolerant SCVs.

Clone CHA strains were isolated worldwide from rivers in Germany, soil in Japan, and from several CF patients in Central Europe [15]. In addition to the original strain used in this study, two other clone CHA strains, one isolated from the environment and another from the sputum of a CF patient with normal lung function, were sequenced [48]. Interestingly, only the here-characterized CHA strain harbors a *gacS* deletion that affects so strongly its virulence properties. Genome examination did not highlight any other genetic event that could explain such deregulation [48]. This indicates that strains of the same clonal group isolated from diverse environments do not necessarily share the same infective properties [48]. Analysis of numerous CF strains with the same T6SS (absence)/T3SS (presence) pattern of expression will help to determine whether this genetic mechanism of turning a bacterium to hypervirulence is an isolated phenomenon or if it is a widespread alternative among CF lung isolates.

The CHA strain over-expresses *alg* genes and overproduces alginate [16]; this mucoid phenotype is due to one mutation in *mucA* leading to replacement of Ala-5 residue by Gly in the anti-sigma MucA protein [48] (Figure S2). Several reports indicated that *mucA* mutation negatively affects flagellum motility [11], [65], leads to a reduced expression of T3SS [66] as well as of other traits of acute virulence like elastase production (T2SS) [67], suggesting that mucoidy correlates with reduced virulence [68], [69]. However, despite a mucoid phenotype, CHA is endowed with efficient T3SS and also motility appendages that may contribute to its aggressive phenotype. Two mechanisms coupling *mucA* mutation to reduced T3SS gene expression have been reported, one dependent and the other independent of the regulator Vfr [47], [68]. Indeed, the cAMP/Vfr-dependent signalling (CVS) pathway, known to activate synthesis of the virulence factors associated with acute disease such as the T3SS, was shown to be turned-off in *mucA*-mutant strains by a mechanism involving AlgU and AlgR; this pathway thus activates alginate synthesis and reduces T3SS-dependent virulence [68]. However, for a still unknown reason, inactivation of *vfr* does not affect T3SS in CHA (B. Toussaint, personal communication); this is surprising in regard to the high induction of CHA T3SS expression in response to calcium depletion, known to induce cAMP synthesis and, consequently, the CVS pathway [70]. Hence, neither the Gac/Rsm pathway nor the CVS pathway modulates the highly active T3SS in this isolate. The second mechanism linking mucoidy to T3SS expression implies a Vfr-independent pathway, in which *mucA* inactivation triggers RsmYZ transcription through activation of AlgZ/AlgR TCS, in a mechanism requiring GacS/GacA TCS [47]. In CHA strain, in which both *mucA* and *gacS* genes are inactive, this particular regulatory pathway can not control T3SS gene expression. Besides these pathways, other elements influence expression of virulence factors involved in acute and persistent infections, such as the second messenger c-di-GMP, which has been reported to inversely control T6SS and T3SS expression [71]. Interestingly, the c-di-GMP dependent-switch is linked, at an unknown level, to the RetS/Gac/Rsm pathway and requires the regulatory sRNAs. As no efficient amounts of sRNAs are synthesized in CHA, the role of c-di-GMP in the regulation of virulence factors of CHA requires additional analyses. Finally, a recent finding has added another level of complexity to the RetS/Gac/Rsm pathway. Indeed, a new member of the RsmA/CsrA family, called RsmF/RsmN, has been identified in *P. aeruginosa* [72], [73]. This new post-transcriptional regulator controls some of RsmA targets, notably T3SS and H1-T6SS mRNA, and its expression is negatively controlled by RsmA. Assessing its role in the virulence pattern of the CHA strain would be another challenge.

To conclude, these data clearly establish that adaptation in CF lungs generates clones with profound deregulation in the intertwined pathways controlling pathogenicity. Their study can allow identifying the different molecular links connecting the essential regulatory cascades and, thus, new therapeutic targets.

## Supporting Information

Figure S1 **Survival rates and bacterial dissemination in CHA-infected mice.** Acute pneumonia was provoked in mice by nasal instillation of a bacterial suspension (5×10^6^ CFU) of either CHA or PAO1. The reference PAO1 strain was provided by A. Rietsch as PAO1F (A) Kaplan-Meyer survival curves were established from 10 infected mice per strain. Statistical differences were calculated with LogRank test. (B) Mice were euthanized 15 hours post-infection; blood and spleen were withdrawn and *P. aeruginosa* CFU were determined in each tissue. Data represent the mean CFU + SEM calculated for total tissue (n = 5 mice per strain). Statistical differences between strain dissemination: p = 0.009 (*) in blood and spleen as established by Mann-Whitney test.
(TIF)

Figure S2 **The mucoid phenotype of CHA is complemented by a functional** ***mucA*** **copy.** CHA strains containing either pJN105 (empty vector) or pJN-MucA were plated on PIA plates containing gentamycin (400 µg/ml) and 0.2% arabinose, as indicated, for 16 h at 37°C.
(TIF)

Materials and Methods S1 **Used for generating Figures S1 and S2.**
(DOC)

Table S1 **Oligonucleotides used in this work.**
(DOC)
