# Supplementary figures and images for: A gacS Deletion in Pseudomonas aeruginosa Cystic Fibrosis Isolate CHA Shapes Its Virulence
Source: PLoS One. 2014 Apr 29;9(4):e95936. doi: 10.1371/journal.pone.0095936 (PMC4004566; doi:10.1371/journal.pone.0095936)

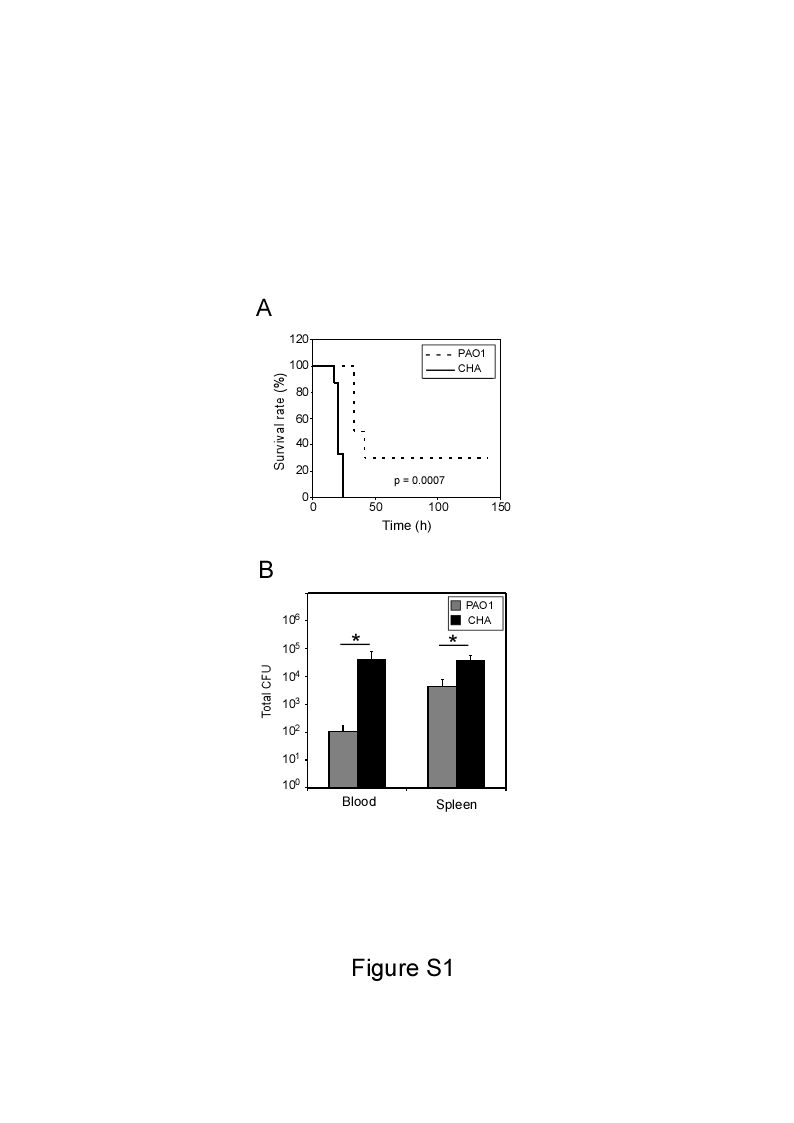

Supplement: Figure S1 — Survival rates and bacterial dissemination in CHA-infected mice. Acute pneumonia was provoked in mice by nasal instillation of a bacterial suspension (5×10^6^ CFU) of either CHA or PAO1. The reference PAO1 strain was provided by A. Rietsch as PAO1F (A) Kaplan-Meyer survival curves were established from 10 infected mice per strain. Statistical differences were calculated with LogRank test. (B) Mice were euthanized 15 hours post-infection; blood and spleen were withdrawn and P. aeruginosa CFU were determined in each tissue. Data represent the mean CFU + SEM calculated for total tissue (n = 5 mice per strain). Statistical differences between strain dissemination: p = 0.009 (*) in blood and spleen as established by Mann-Whitney test. (TIF) [file pone.0095936.s001.tif]

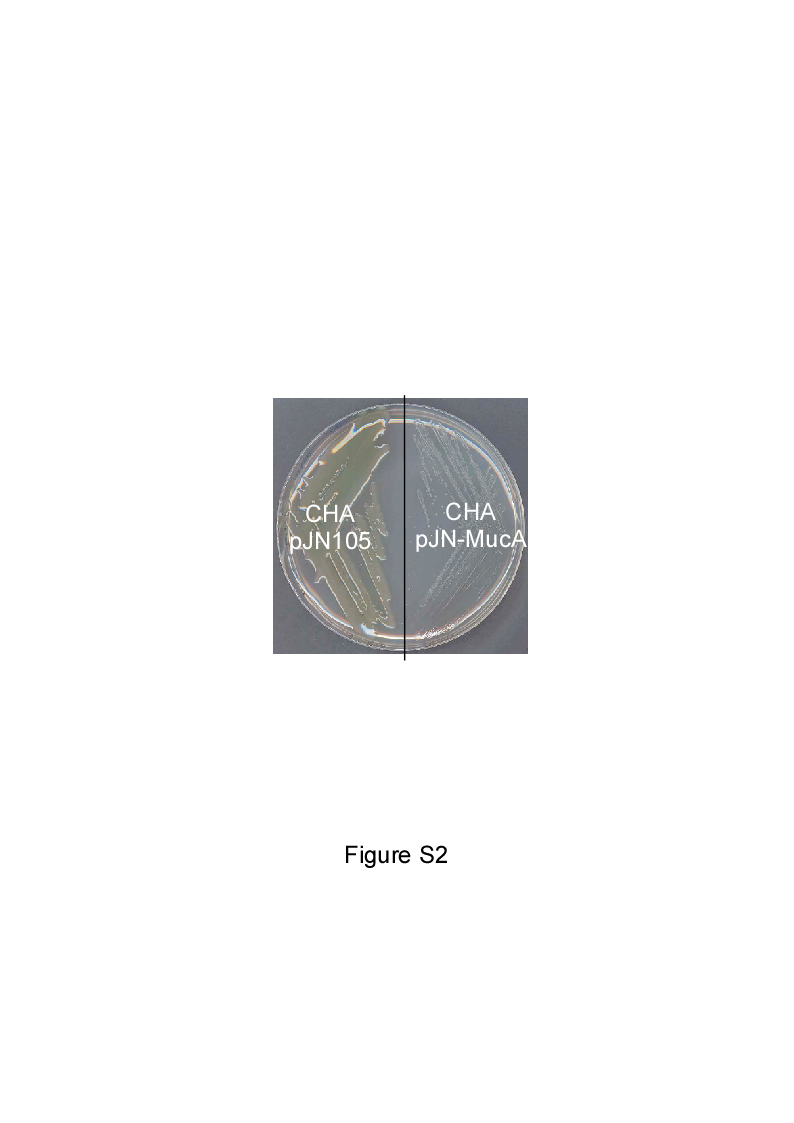

Supplement: Figure S2 — The mucoid phenotype of CHA is complemented by a functional mucA copy. CHA strains containing either pJN105 (empty vector) or pJN-MucA were plated on PIA plates containing gentamycin (400 µg/ml) and 0.2% arabinose, as indicated, for 16 h at 37°C. (TIF) [file pone.0095936.s002.tif]
