# Supplementary material for: A gacS Deletion in Pseudomonas aeruginosa Cystic Fibrosis Isolate CHA Shapes Its Virulence
Source: PLoS One. 2014 Apr 29;9(4):e95936. doi: 10.1371/journal.pone.0095936 (PMC4004566; doi:10.1371/journal.pone.0095936)
Supplement: Materials and Methods S1 — Used for generating Figures S1 and S2. (DOC) [file pone.0095936.s003.doc]

**PONE –D-14-00707**

**A *gacS* deletion in *Pseudomonas aeruginosa***

**cystic fibrosis isolate CHA shapes its virulence**

Khady Mayebine Sall, Maria Guillermina Casabona, Christophe Bordi, Philippe Huber, Sophie de Bentzmann, Ina Attrée, Sylvie Elsen

**Supplemental Materials and Methods**

**Genetic construction**

For *mucA* complementation, a 657 bp region encompassing the entire *mucA* sequence was amplified by PCR from PAO1 genomic DNA using appropriate primer pairs (see Supplemental Table 1), cloned into pCR-Blunt II-TOPO and sequenced. The *Eco*RI-*Xba*I fragment was then excised and cloned into *Eco*RI*-Xba*I-cut pJN105. The resulting vector was introduced in *P. aeruginosa* by transformation.

**Animals**

All protocols in this study were conducted in strict accordance with the French guidelines for the care and use of laboratory animals. The protocol for mouse infection was approved by the animal research committee of the institute (CETEA). Pathogen-free BALB/c male mice (8-10 weeks) were obtained from Harlan Laboratories and housed in the CEA animal care facilities.

***P. aeruginosa*-induced lung injury**

Bacteria were grown to an optical density of 1.0 at 600 nm (*A_600_*) in LB, at 37°C. They were centrifuged and resuspended in sterile PBS at 1.7 x 10^8^ per ml as evaluated by spectrometry. Mice were anesthetized by intraperitoneal administration of a mixture of xylazine (10 mg/kg) and ketamine (50 mg/kg). Then, 30 µl of bacterial suspension (*i.e.* 5 x 10^6^ bacteria) were deposited into mouse nostrils. For survival curves, time of mouse death was noted and presented as Kaplan-Meyer curves (n = 10 per condition). Statistical differences were established by Log-Rank test. For investigation of bacterial dissemination (n = 5 per condition), mice were euthanized by CO_2_ inhalation 15 h later. Blood was withdrawn in the heart and spleens were dissected and homogenized with a Polytron in 2 ml PBS. Colony-forming units in each tissue were counted after serial dilutions and spreading on PIA plates. In uninfected mice, no CFU were observed. Statistical differences were established by Mann-Whitney test.
