# Supplementary material for: A gacS Deletion in Pseudomonas aeruginosa Cystic Fibrosis Isolate CHA Shapes Its Virulence
Source: PLoS One. 2014 Apr 29;9(4):e95936. doi: 10.1371/journal.pone.0095936 (PMC4004566; doi:10.1371/journal.pone.0095936)
Supplement: Table S1 — Oligonucleotides used in this work. (DOC) [file pone.0095936.s004.doc]

**PONE –D-14-00707**

**A *gacS* deletion in *Pseudomonas aeruginosa***

**cystic fibrosis isolate CHA shapes its virulence**

Khady Mayebine Sall, Maria Guillermina Casabona, Christophe Bordi, Philippe Huber, Sophie de Bentzmann, Ina Attrée, Sylvie Elsen

**Table S1.** Oligonucleotides used in this work

| Primers | DNA sequences^a^ |
| --- | --- |
| *rsmA* mutagenesis |  |
| Mut-RsmA-F1 | 5’-CCCGGGAAGCCATCGGCGACACCAAC-3’(*Sma*I) |
| Mut-RsmA-R1 | 5’-CCGGTGTACGGCGACTTCTCACATTCCTTTCTCCTCACGCGAA |
| Mut-RsmA-F2 | 5’-GAAGTCGCCGTACACCGGGAG-3’ |
| Mut-RsmA-R2 | 5’-CCCGGGAGGATTCGAACCTCCGACC-3’(*Sma*I) |
| *rsmA* complementation |  |
| PSE-RsmA-F | 5’-GGTCTAGAATATTCGCGTGAGGAGAAAGG-3’(*Xba*I) |
| PSE-RsmA-R | 5’-CCAAGCTTAATTAATGGTTTGGCTCTTGATC-3’(*Hin*dIII) |
| *retS* mutagenesis |  |
| RetS-F1 | 5’-CCCGGGACTTCGCCGTGGTACGG-3’  (*Sma*I) |
| RetS-R1 | 5’-GAAGTCGCTCGGCAGGCTCGCGACTCGCCGGTGTGC-3’ |
| RetS-F2 | 5’-GAGCCTGCCGAGCGACTTCC-3’ |
| RetS-R2 | 5’-CCCGGGCAGGGCGTCGCCCTG-3’(*Sma*I) |
| *gacS* complementation |  |
| - pJN105 |  |
| GacS-SmaI-F | 5’-CCCGGGGAGCGTCGGCCATC-3’ (*Sma*I) |
| GacS-XbaI-R | 5‘-TCTAGAAGAACAGGATGCGCATGGTC-3’ (*Xba*I) |
| - mini-TN7T |  |
| P-GacS-F | 5’-CCCCCGGGCAGTGTCGGATCGTCCTCGAT-3’ (*Sma*I) |
| P-GacS-R | 5’-CCAAGCTTGGTCAGAGTTCGCTGGAGTCG-3’ (*Hin*dIII) |
| *mucA* complementation |  |
| mucA-EcoRI-F | 5’-GAATTGCACAGCGGCAAATGCCAA-3’ |
| mucA-XbaI-R | 5’-TCTAGACAACAGGGAGGTGGTGCGCA-3’ (*Xba*I) |
| VSV-G Tag (PAO1) |  |
| GacS VSV-G-F1 | 5’-GCCCGGGCCGCAGCTCGGCCAGTTGA-3’(*Sma*I) |
| GacS VSV-G-R1 | 5’-TTTTCCTAATCTATTCATTTCAATATCTGTATAGAGTT |
|  | CGCTGGAGTCGAGGCT -3’ |
| GacS VSV-G-F2 | 5’-TATACAGATATTGAAATGAATAGATTAGGAAAATGAC |
|  | CATGCGCATCCTGTTCTTC-3’ |
| GacS VSV-G-R2 | 5’-GCCCGGGCGGGTCCGGTTGTAGGCGC-3’(*Sma*I) |
| VSV-G Tag (CHA) |  |
| CHAGacS VSV-G-F1 | 5’-GCCCGGGTAGTGCTGAAGTGGACCGG-3’(*Sma*I) |
| CHAGacS VSV-G-R1 | 5’-TTTTCCTAATCTATTCATTTCAATATCTGTATAGGCCG |
|  | ACCGCGTGCTCGG-3’ |
| CHAGacS VSV-G-F2 | 5’-TATACAGATATTGAAATGAATAGATTAGGAAAATGAT |
|  | CCTGACGCTCAACCGGC-3’ |
| CHAGacS VSV-G-R2 | 5’-GCCCGGGATCAGCATCGCGCCGGGTTT-3’(*Sma*I) |
| RT-PCR |  |
| 16s RNA up | 5’-CAGCTCGTGTCGTGAGATGT-3’ |
| 16s RNA down | 5’-GATCCGGACTACGATCGGTT-3’ |
| gacS up | 5’-CACGAGAAGCAACTGGAACTG-3’ |
| gacS down | 5’-CTG GCTGAAGGCCTTGAACAA-3’ |
| RT-qPCR |  |
| rsmYUp | 5’-AGGACATTGCGCAGGAAG-3’ |
| rsmYDn | 5’-GGGGTTTTGCAGACCTCTC-3’ |
| rsmZUp | 5’-CGTACAGGGAACACGCAAC-3’ |
| rsmZDn | 5’-GTATTACCCCGCCCACTCTT-3’ |
| pelAUp | 5’-CAGGTGCTGGAGGACTTCAT-3’ |
| pelADn | 5’-AGAACGGATGGCTGAAGGTA-3’ |
| exoSUp | 5’-CTCTACACCGGCATTCACTA-3’ |
| exoSDn | 5’-CTTCACTACCTGTTCAGCCT-3’ |
| PA0094up | 5’-Ccatttctacgactggcaca-3’ |
| PA0094do | 5’-gggtagtcgtacaagcg-3’ |

^a^Restriction sites incorporated into primers are underlined and indicated in brackets
